# Factors Influencing Human Immunodeficiency Virus Testing Results in Mozambique: A Multivariate Approach to Socioeconomic, Demographic, and Cultural Influences

**DOI:** 10.1002/hsr2.72148

**Published:** 2026-04-22

**Authors:** Md. Rasel Hossain, Kabir Hossain, Riyadh Hossain, Indi Islam, Nazia Akter, Md. Owaliur Rahman Akanda

**Affiliations:** ^1^ Department of Statistics Noakhali Science and Technology University Noakhali Bangladesh; ^2^ Department of Law Islamic University Kushtia Bangladesh; ^3^ Department of Education Noakhali Science and Technology University Noakhali Bangladesh

**Keywords:** demographic and cultural determinants, demographic and health survey, HIV/AIDS, Mozambique, socioeconomic

## Abstract

**Background:**

HIV/AIDS has been a significant health issue among the population in Mozambique, Sub‐Saharan Africa. Although there are studies in the region that have investigated the determinants of HIV testing, very few have covered Mozambique with current and comprehensive information. This research was aimed at establishing the socioeconomic, demographic, and cultural factors that had been associated with an HIV test result and provide an up‐to‐date assessment of the dynamic change in the HIV risk in the nation.

**Methods:**

This study explored the favorable association between the results of the HIV tests and the selected background factors based on the 3729 respondents who participated in the 2022–2023 Mozambique Mini Demographic and Health Survey (MMDHS). The data were analyzed using Chi‐square and binary logistic regression techniques and the SPSS version 25. Model fit was assessed by the Hosmer–Lemeshow test.

**Result:**

The odds of testing positive for HIV increased with age, with individuals aged 26–30 (OR = 3.60, CI: 1.95), 31–35 (OR = 4.74, CI: 2.56), and over 35 years (OR = 7.57, CI: 4.16) showing higher likelihoods compared to those aged 15–20. Respondents living in rural areas (OR = 0.75, CI: 0.57–0.99) and those with secondary (OR = 0.51) or higher education (OR = 0.13) were less likely to test positive. Regarding relationship type, respondents with “live‐in partners” had lower odds of HIV infection (OR = 0.708), whereas those in the “Others” category had substantially higher risk (OR = 2.135). Higher odds were significantly associated with genital ulcers (OR = 1.81) and having more than three sexual partners in the past year (OR = 1.99).

**Conclusion:**

The analyses reveal the important links between the status of HIV and socioeconomic, demographic, and cultural factors in Mozambique. It highlights the importance of targeted interventions that encourage safe sex and more education programs, as well as better awareness of HIV and increased access to testing among different population groups.

**Abbreviations**
AIDS: acquired immunodeficiency syndrome
ART: antiretroviral therapy/odds ratio
CI: confidence interval
HIV: human immunodeficiency virus
MMDHS: Mozambique mini demographic and health survey
OR: odds ratio
PTSD: posttraumatic stress disorder
STIs: sexually transmitted infections
UNICEF: United Nations International Children's Emergency Fund
USAID: United States Agency for International Development

## 1 Introduction

The human immunodeficiency virus (HIV) is a pathogen that damages the immune system by targeting white blood cells, making the body increasingly susceptible to infections and diseases. The advanced stage of HIV infection, known as acquired immunodeficiency syndrome (AIDS), is often initiated by the transmission of the virus through contaminated bodily fluids, including blood, breast milk, semen, or vaginal secretions. Antiretroviral therapy (ART) effectively manages HIV by inhibiting the virus's replication and diminishing the likelihood of transmission to others. This treatment effectively addresses critical public health issues and empowers individuals to maintain healthy lifestyles [1]. However, the stigma associated with HIV continues to pose a substantial barrier that impedes timely access to treatment and adherence to ART, consequently undermining initiatives aimed at controlling the epidemic [2]. Therefore, the increasing incidence and death rates associated with HIV/AIDS globally are alarming, demonstrating the ongoing difficulties in effectively addressing the epidemic, regardless of the progress made in prevention and treatment methods [3]. In addition, mental health concerns, including anxiety [4], depression [5], suicidal ideation, posttraumatic stress disorder (PTSD), societal stigma, and psychological distress [6], are significantly more prevalent among people living with HIV/AIDS (PLWHA).

Worldwide, millions of individuals are affected by HIV, and each year, a significant number of new infections and deaths are reported, highlighting the epidemic as a pressing public health concern, it was estimated that approximately 39.9 million individuals worldwide were living with HIV. In the previous year, there were also 630,000 deaths attributed to HIV‐related causes and 1.3 million newly documented cases of HIV [7]. The prevalence and effects of the global HIV/AIDS epidemic varied significantly by region between 1990 and 2019. The burden was still greatest in Africa, where the prevalence rate in 2019 was 1.99%. In contrast, rates in the Americas, Asia, and Europe were significantly lower, at 0.39%, 0.1%, and 0.254%, respectively [8]. Mozambique is one of the countries in Eastern and Southern Africa where HIV is most prevalent, recording an estimated 2.1 million people living with the virus and a 12.5% prevalence rate in 2017. Additionally, 130,000 new infections and 70,000 AIDS‐related deaths were reported nationwide [9]. A recent study also revealed that the prevalence of HIV has significantly increased among Mozambican men aged ≥ 18 who have sex with men (MSM), underscoring their increasing vulnerability [10]. It is estimated that the number of HIV/AIDS cases will continue to rise gradually over time [11].

There are many interrelated factors that affect the prevalence, course, and treatment of HIV/AIDS. Gender‐based disparities in vulnerability exist, highlighted by the fact that women are, on average, 60% more likely than men to contract HIV [12]. Additionally, the prevalence of HIV infection varies significantly by region, with middle‐aged populations having the highest rates [13]. HIV prevalence among people aged 15 to 24 in Eastern and Southern Africa is at least 1.5 times higher in cities than in rural areas [14]. Additionally, educational attainment is important because HIV prevalence is concentrated among those with less education [15]. HIV risk is influenced differently by socioeconomic status (SES) for men and women. While lower SES is associated with fewer HIV tests for men, it is also related to increased sexual risk‐taking behaviors for women, which makes them even more vulnerable [16]. Environmental and behavioral factors also play a major role in HIV transmission. For example, the main modes of HIV transmission in China and the US are heterosexual sex and MSM [17]. Meanwhile, injection drug use, sharing of needles, and a history of incarceration are strongly associated with HIV infection due to the increased risk of encountering contaminated blood [18]. In Mozambique, the general population has an HIV prevalence of 12.5%, but people who inject drugs have a prevalence of almost 50% [19]. For MSM who had two sexual partners in the last year, the HIV prevalence increases from 5.2% to 14.4% [10]. Premarital and extramarital sex contributes to a higher adolescent fertility rate, which in turn raises the risk of HIV infection by resulting in longer periods of sexual activity [20].

Demographic and cultural factors also play a critical role. In Africa, Islamic cultural or behavioral practices have been associated with a lower prevalence of HIV than Christian populations [21, 22]. However, factors such as living in multiple locations and never having children are significant risk factors for women [23]. Meanwhile, a lack of knowledge about HIV and sexually transmitted infections (STIs) leads to risky behaviors, delayed treatment, and higher transmission rates among HIV‐positive people [24]. Increasing awareness of STIs and encouraging consistent condom use are essential ways to mitigate the spread of HIV [25]. Recent genital symptoms like discharge, knowledge and use of HIV test kits, and the ability to talk about condom use with partners are all associated with HIV testing among women of reproductive age [26].

There are still important research gaps despite tremendous advancements in our understanding of HIV testing uptake and infection dynamics in Africa. Although previous research has provided valuable regional insights, such as analyzing predictors for HIV testing utilizing demographic and health survey data from 2015 to 2022 across sub‐Saharan Africa, it has not explicitly addressed Mozambique due to issues with the dataset's completeness [27]. In a similar vein, the 2015 Vaccination, Malaria, and HIV/AIDS Indicators Survey conducted in Mozambique identified sociodemographic factors related to HIV among adolescents aged 15 to 24. However, its limited variables, outmoded data, and age‐specific focus restricted it from investigating the broader determinants of HIV testing outcomes [28].

This study employs the updated Mozambique Mini Demographic and Health Survey (MMDHS) for 2022–2023 to mitigate these limitations, considering a range of socioeconomic, demographic, and cultural variables. Specifically, this study examines the factors influencing both positive and negative HIV test results through an analysis of a more recent and extensive dataset. This analysis also reflects the changing dynamics associated with HIV testing. Compared to earlier studies, including a varied range of independent variables significantly improves the capacity to evaluate the interactions among determinants and provides a more comprehensive perspective.

## 2 Material and Methods

### 2.1 Study Area and Data Collection

This secondary analysis used data from the 2022–2023 MMDHS to examine factors influencing the prevalence of HIV and AIDS in Mozambique, a Southern African country covering 801,537 square kilometers (25°57′ S; 32°35′ E) [29, 30]. It is bordered by South Africa and Eswatini to the south, Zimbabwe and Zambia to the west, Malawi to the north, and Tanzania to the north. Maputo is the capital and port city of Mozambique. It is situated on the northern border of the Espírito Santo Estuary in Delagoa Bay, an inlet of the Indian Ocean [31]. Mozambique is the thirteenth most populous country in Africa [32]. The data for this investigation were obtained from the respondent's records of the 2022–2023 MMDHS [33, 34]. The Institute of National Statistics implemented the Demographic and Health Survey 2022–2023 in Mozambique (IDS 2022–2023). IDS 2022–2023 was funded by the Government of Mozambique, the United States Agency for International Development (USAID), the World Bank, UNICEF, the Foreign, Commonwealth & UK Development Office (FCDO), the Canadian High Commission, and Gavi, the Vaccines Alliance. ICF provided technical assistance through the DHS Program, a USAID‐funded project that provides support and technical assistance in implementing demographic and health surveys in countries worldwide [34].

### 2.2 Study Population

A total of 3729 respondents were included in the 2022–2023 MMDHS HIV and AIDS recorded data, with 573 of them was taken last HIV and AIDS test report among 12.60% tested positive at government hospital and 2897 of them was taken last HIV and AIDS test report among 12.20% tested positive at health center and 259 of them was taken last HIV and AIDS test report among 4.20% tested positive at others private sector. The research population of this study consisted of 3729 respondents’ born in a clinical setting in Mozambique. These respondents were questioned regarding HIV and AIDS, and 11.7% (*n* = 435) of them were discovered to have HIV and AIDS (Table 1). Missing data were examined before analysis, and variables with minimal missingness were removed using listwise deletion. As no variable exceeded the acceptable threshold, imputation was not needed. Analyses were conducted on the complete‐case dataset.

**Table 1:** Percentage distribution of the respondents by background characteristic for the Result of HIV test in Mozambique.

Variables	Categories	Frequency	Percent
Region	North	603	16.2
	Central	846	22.7
	South	2280	61.1
Respondent's current age	15–20	649	17.4
	21–25	799	21.4
	26–30	706	18.9
	31–35	546	14.6
	> 35	1029	27.6
Type of place of residence	Urban	2250	60.3
	Rural	1479	39.7
Highest educational level	No education	366	9.8
	Primary	1331	35.7
	Secondary	1785	47.9
	Higher	247	6.6
Religion	Catholic	689	18.5
	Islamic	342	9.2
	Zion	662	17.8
	Evangelical/Pentecostal	1700	45.6
	Others	336	9
Socio‐economic status	Poor	657	17.6
	Middle	585	15.7
	Rich	2487	66.7
Heard about other STIs	No	1046	28.1
	Yes	2683	71.9
Births in last 5 years	No births	1640	44
	1	1622	43.5
	> 1	467	12.5
Ever heard of a STI	No	1046	28.1
	Yes	2683	71.9
Condom used during last sex with most recent partner	No	2675	71.7
	Yes	1054	28.3
Had any STI in last 12 months	No	3364	90.2
	Yes	365	9.8
Had genital sore/ulcer in last 12 months	No	3490	93.6
	Yes	239	6.4
Had genital discharge in last 12 months	No	3047	81.7
	Yes	682	18.3
Number of sex partners, including spouse, in last 12 months	1	3460	92.8
	> 1	269	7.2
Relationship with most recent sex partner	Spouse	1316	35.3
	Boyfriend not living with respondent	1161	31.1
	Live‐in‐partner	1024	27.5
	Others	228	6.1
Wife justified asking husband to use condom if he has STI	No	1073	28.8
	Yes	2656	71.2
Drugs to avoid HIV transmission to baby during pregnancy	No	515	13.8
	Yes	3214	86.2
Would buy vegetables from vendor with HIV	No	629	16.9
	Yes	3100	83.1
Months ago most recent HIV test	0–12	1967	52.7
	> 12	1762	47.3
Place where last HIV test was taken	Government Hospital	573	15.4
	Health center	2897	77.7
	Others	259	6.9
Total lifetime number of sex partners	1	1036	27.8
	2–3	1637	43.9
	> 3	1056	28.3
Knowledge and use of HIV test kits	Never heard of HIV test kits	2971	79.7
	Has tested with HIV test kits	180	4.8
	Knows test kits but never tested with them	578	15.5
Heard of ARVs to treat HIV	No	242	6.5
	Yes	3487	93.5
Children with HIV should be allowed to attend school with children without HIV	No	519	13.9
	Yes	3210	86.1
Month of most recent HIV test	1–6	1569	42.1
	7–12	1501	40.3
	Don't know	659	17.7
Year of most recent HIV test	2003–2020	1005	27
	2021	820	22
	> 2021	1904	51.1
Method used last sexual intercourse	No	275	7.4
	Yes	3454	92.6
Result of HIV test	Negative	3294	88.3
	Positive	435	11.7
Number of HIV tests	1–2	929	24.9
	3–4	1191	31.9
	> 4	1609	43.1

### 2.3 Dependent Variables

The binary variable “Result of HIV test” (Negative, Positive) was the dependent variable in this study. Respondents who effected at least one time with HIV and AIDS were classified as “Positive,” while those who did not affected with HIV and AIDS were designated as “Negative.”

### 2.4 Explanatory Variables

This study has considered a total of 22 independent variables. Variables were selected based on theoretical relevance and prior evidence from the literature. Three distinct categories of explanatory variables were used: socioeconomic, demographic, and cultural characteristics. Socioeconomic variables encompass educational levels (No education, Primary, Secondary, and Higher), place of residence (Urban, Rural), and SES (Poor, Middle, and Rich).

The demographic group is comprised of the following: Respondent's current age (15–20, 21–25, 26–30, 31–35, > 35), ever heard of a STI (no, yes), Births in last 5 years (no births, 1, > 1), condom used during last sex with most recent partner (no, yes), had any STI in last 12 months (no, yes), Had genital sore/ulcer in last 12 months (no, yes), Had genital discharge in last 12 months (no, yes), number of sex partners, including spouse, in last 12 months (1, > 1), relationship with most recent sex partner (Spouse, Boyfriend not living with respondent, Live‐in‐partner, Others), wife justified asking husband to use condom if he has STI (no, yes), drugs to avoid HIV transmission to baby during pregnancy (no, yes), place where last HIV test was taken (Government Hospital, Health center, Others), total lifetime number of sex partners (1, 2–3, > 3), knowledge and use of HIV test kits (Never heard of HIV test kits, Heard of ARVs to treat HIV (no, yes), method used last sexual intercourse (no, yes), Number of HIV tests (1 – 2, 3 – 4, > 4). Finally, cultural features were the religion (Catholic, Islamic, Zion, Evangelical/Pentecostal, and others) and region (North, Central, South).

At first, the respondent's current age, Births in the last 5 years, Number of sex partners, including spouse, in the last 12 months, Total lifetime number of sex partners, and Number of HIV tests were discrete numeric variables. However, as previously mentioned, they were converted into meaningful categories based on their natural distribution, established cut‐offs in prior literature, and to enhance interpretability within a logistic regression framework. In addition, key socioeconomic and demographic variables were included in the analysis to control for potential confounding effects, following DHS analytical guidelines. This approach ensured more accurate and unbiased estimates of associations.

### 2.5 Statistical Analysis

The data for this investigation were obtained from the 2022–2023 MMDHS, with a total of 3729 respondents. Among them, 3294 (88.3%) tested HIV‐negative, while 435 (11.7%) tested HIV‐positive. Multicollinearity was assessed using variance inflation factors (VIF), and stepwise selection was applied as needed to reduce redundancy and prevent overfitting, while retaining variables of clinical or theoretical importance.

Frequencies of all independent and dependent variables were examined to describe respondent characteristics. Nonparametric methods were applied due to the categorical nature of the outcome variable. The Chi‐square (*χ*²) test was used to assess associations between HIV status and independent variables. Variables significant in the bivariate analysis were included in the multivariate logistic regression. Both bivariate and multivariate analyses were evaluated at a 5% significance level (*p* < 0.05). Model fit was assessed using the Hosmer‐Lemeshow test (*χ*² = 13.09, *p* = 1.09), indicating an appropriate fit. All analyses were performed using SPSS version 25.

### 2.6 Ethical Approval and Informed Consent

According to the ethical principles described in the Declaration of Helsinki, the authors undertook all requisite measures to comply with the established guidelines for medical research. This study used publicly available secondary data from the 2022–2023 MMDHS, conducted through The DHS Program. The MMDHS 2022–2023 followed standardized and internationally accepted ethical procedures for data collection, and informed consent was obtained from all respondents prior to their participation in the survey. The survey protocol received ethical approval from the relevant national ethics authorities in Mozambique as well as the Institutional Review Board (IRB) of ICF. Since the present study relied solely on anonymized secondary data and did not involve direct interaction with participants or the collection of new information, additional ethical approval was not required. However, permission to access and use the 2022–2023 MMDHS dataset for this analysis was obtained from The DHS Program.

## 3 Result

**Figure 1:**
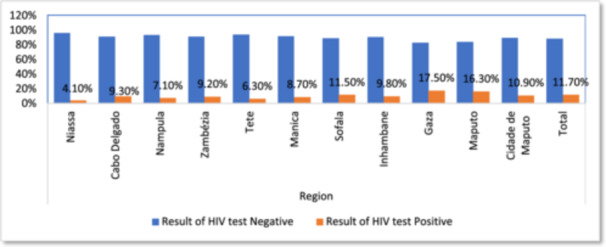
The percentage of Demographic results for HIV and AIDS in various demographic regions of Mozambique.

The prevalence of HIV in Mozambique varies by region, as illustrated in Figure 1. The Northern provinces, notably Niassa (4.1%), Nampula (7.1%), and Cabo Delgado (9.3%), had the lowest positivity rates, indicating a lower HIV burden despite poor healthcare access in remote areas. The Central area has a moderate frequency, with rates ranging from 6.3% in Tete to 11.5% in Sofala, most likely reflecting variations in healthcare availability, education, and test coverage. In contrast, the Southern area has the highest prevalence, particularly in Gaza (17.5%) and Maputo Province (16.3%), followed by Maputo City (10.9%), which could be attributed to greater urbanization, population movement, and high‐risk behaviors. Overall, the national HIV positivity rate is 11.7%, indicating significant geographical differences.

**Figure 2:**
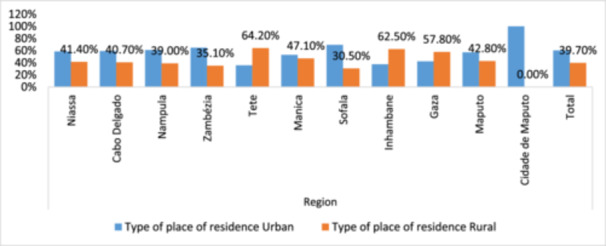
The ratio of results for HIV and AIDS in urban and rural contexts across various regions of Mozambique.

Figure 2 illustrates the disparities in HIV positivity between urban and rural areas throughout Mozambique. Rural regions in the North exhibit a higher prevalence of HIV, recorded at 10.3%, in comparison to urban areas, which have a rate of 7.3%. The Central region demonstrates a similar trend, with rural areas (9.3%) marginally surpassing urban areas (8.9%), indicating potential disparities in healthcare access and instances of underreporting. Urban regions in the South, accounting for 12.6%, demonstrate the highest positivity rate, a phenomenon attributable to elevated‐risk behaviors, migration patterns, and increased population density. Rural regions (10.3%) continue encountering substantial obstacles to healthcare and testing, resulting in underdiagnosis and inadequate prevention initiatives.

**Figure 3:**
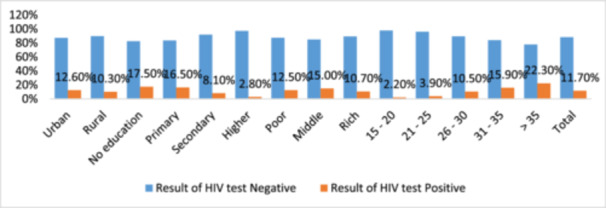
The results for HIV and AIDS in Mozambique indicate the proportion of various demographic characteristics.

In Mozambique, there are substantial disparities in HIV prevalence that are influenced by region, age, education, SES, and sexual behaviors (in Figure 3). The South (13.9%) has the highest prevalence, which is influenced by urbanization and riskier sexual behaviors. Conversely, the North (7.3%) has the lowest prevalence, which suggests that there is a need for enhanced healthcare access.

The most excellent HIV rates are observed in individuals over the age of 35 (22.3%), which implies that there is a lack of awareness and prolonged exposure to risk. Individuals with a higher level of education exhibit reduced rates (2.8%), whereas those uneducated are more susceptible (17.5%) in Figure 3. Individuals with multiple sexual partners and genital lesions exhibit higher positivity rates, while impoverished populations (12.5%) are at a higher risk due to limited access to healthcare.

Table 1 presents the frequency and percentage distribution of respondents across multiple demographics, socio‐economic, behavioral, and HIV‐related characteristics. By region, 16.2% were from the North, 22.7% from the Central, and 61.1% from the South. Age distribution shows 17.4% were aged 15–20, 21.4% were 21–25, 18.9% were 26–30, 14.6% were 31–35, and 27.6% were older than 35. A majority lived in urban areas (60.3%), while 39.7% resided in rural areas. Educational levels included no education (9.8%), primary (35.7%), secondary (47.9%), and higher education (6.6%). Religious affiliation included Catholic (18.5%), Islamic (9.2%), Zion (17.8%), Evangelical/Pentecostal (45.6%), and others (9.0%). Socio‐economic status consisted of poor (17.6%), middle (15.7%), and rich (66.7%). Birth history in the last 5 years shows 44.0% had no births, 43.5% had one birth, and 12.5% had more than one. In general, awareness of STIs follows the similar pattern, with 28.1% unaware and 71.9% aware. Condom use during the last sexual encounter was reported as 71.7% not using and 28.3% using. In the past 12 months, 90.2% reported no STI, and 9.8% reported having one. Genital sores were absent in 93.6% and present in 6.4%, while genital discharge was absent in 81.7% and present in 18.3%. The number of sexual partners in the last 12 months was one for 92.8% and more than one for 7.2%. The relationship with the most recent partner included spouse (35.3%), boyfriend not living with respondent (31.1%), live‐in partner (27.5%), and others (6.1%). Regarding attitudes, 28.8% did not think a wife is justified in asking her husband to use a condom if he has an STI, while 71.2% agreed.

Awareness of drugs to prevent mother‐to‐child transmission was reported by 86.2%, while 13.8% were unaware. The last test was done at a government hospital (15.4%), health center (77.7%), or other locations (6.9%). The lifetime number of sexual partners was 1 for 27.8%, 2–3 for 43.9%, and more than 3 for 28.3%. Awareness and use of HIV self‐test kits included never heard of (79.7%), tested with kits (4.8%), and aware but never tested (15.5%). Awareness of ARVs was high, with 93.5% aware and 6.5% unaware. Method use during the last sexual intercourse was reported by 92.6%, while 7.4% reported no method used. HIV test results showed 88.3% tested negative and 11.7% tested positive. The total number of lifetime HIV tests was 1–2 for 24.9%, 3–4 for 31.9%, and more than 4 for 43.1%.

The bivariate analysis was conducted along with the univariate analysis to investigate the relationship between the results of HIV and AIDS and the various explanatory variables among the respondents in Mozambique. The chi‐square test was implemented to evaluate these associations, and the results are summarized in Table 2. HIV positivity rates differed substantially between areas, with the South having the highest rate (13.9%) compared to the North (7.3%) and Central (8.9%) (*χ*² = 28.258, *p* < 0.05). Positive rates of HIV increased significantly with age, from 2.2% among those aged 15%–20% to 22.3% among those over 35 years old (*χ*² = 226.528, *p* < 0.05). Residence type also demonstrated a significant association, with urban residents showing a slightly higher positivity rate (12.6%) than rural residents (10.3%) (*χ*² = 4.584, *p* < 0.05). Educational level had a pronounced effect on HIV outcomes, as individuals with no education exhibited the highest positivity rate (17.5%), while those with higher education had the lowest (2.8%), suggesting that education plays a critical role in HIV awareness and preventive behaviors (*χ*² = 83.71, *p* < 0.05). Religious affiliation significantly influenced HIV positivity. Zion adherents showed the highest positivity rate (15.4%), followed by Evangelical/Pentecostal groups (13.0%), whereas Catholics and Muslims had lower rates (*χ*² = 30.325, *p* < 0.05). Individuals in the poor group had a higher positivity rate (12.5%) than those in the affluent group (10.7%) (*χ*² = 9.362, *p* < 0.05), suggesting that wealth disparities may impede access to HIV‐related care. A history of genital sores or ulcers in the past year significantly increased HIV positivity (18.4%), highlighting the biological link between STI symptoms and susceptibility to HIV infection (*χ*² = 11.274, *p* < 0.05). There was a significant association between HIV status and partner type (*χ*² = 86.408, *p* < 0.05). Positivity was highest in the “Others” group (30.3%) and lowest among married respondents (5.47%), with intermediate rates for noncohabiting boyfriends (10.0%) and live‐in partners (9.1%). Individuals with multiple sexual partners in the past 12 months also had higher positivity (16.0%) compared to those with only one partner (11.3%), underscoring sexual behavior as a major risk factor (*χ*² = 5.25, *p* < 0.05). Awareness of ARVs presented a significant relationship with HIV outcomes, where those knowledgeable about ARVs showed lower positivity (12.2%) than those unaware (3.3%) (*χ*² = 17.551, *p* < 0.05). The place of the last HIV test was also significantly associated with outcomes, where those tested at government hospitals and health centers exhibited higher positivity compared to other facilities (*χ*² = 14.944, *p* < 0.05). Total lifetime number of sexual partners demonstrated a strong association, as positivity increased from 6.3% among those with one partner to 18.5% among individuals with more than three partners (*χ*² = 78.126, *p* < 0.05).

**Table 2:** Cross‐tabulation and associated summary statistics for the result of HIV test in Mozambique.

Variables	Categories	Result of HIV test		Chi‐square	*p* value
		Negative (%)	Positive (%)		
Region	North	559 (92.70)	44 (7.30)		
	Central	771 (91.10)	75 (8.90)	28.258	0
	South	1964 (86.10)	316 (13.90)		
Respondent's current age	15–20	635 (97.80)	14 (2.20)		
	21–25	768 (96.10)	31 (3.90)		
	26–30	632 (89.50)	74 (10.50)	226.528	0
	31–35	459 (84.10)	87 (15.90)		
	> 35	800 (77.70)	229 (22.30)		
Type of place of residence	Urban	1967 (87.40)	283 (12.60)		
	Rural	1327 (89.70)	152 (10.30)	4.584	0.032
Highest educational level	No education	302 (82.50)	64 (17.50)		
	Primary	1111 (83.50)	220 (16.50)		
	Secondary	1641 (91.90)	144 (8.10)	83.71	0.000
	Higher	240 (97.20)	7 (2.80)		
Religion	Catholic	640 (92.90)	49 (7.10)		
	Islamic	314 (91.80)	28 (8.20)	30.325	0
	Zion	560 (84.60)	102 (15.40)		
	Evangelical/Pentecostal	1479 (87.00)	221 (13.00)		
	Others	301 (89.60)	35 (10.40)		
Socio economic status	Poor	575 (87.50)	82 (12.50)		
	Middle	497 (85.00)	88 (15.00)	9.362	0.009
	Rich	2222 (89.30)	265 (10.70)		
Heard about other STIs	No	931 (89.00)	115 (11.00)		
	Yes	2363 (88.10)	320 (11.90)	0.635	0.425
Births in last 5 years	No births	1430 (87.20)	210 (12.80)		
	1	1438 (88.70)	184 (11.30)	6.004	0.05
	> 1	426 (91.20)	41 (8.80)		
Ever heard of a STI	No	931 (89.00)	115 (11.00)		
	Yes	2363 (88.10)	320 (11.90)	0.635	0.425
Condom used during last sex with most recent partner	No	2371 (88.60)	304 (11.40)		
	Yes	923 (87.60)	131 (12.40)	0.831	0.362
Had any STI in last 12 months	No	2981 (88.60)	383 (11.40)		
	Yes	313 (85.80)	52 (14.20)	2.616	0.106
Had genital sore/ulcer in last 12 months	No	3099 (88.80)	391 (11.20)		
	Yes	195 (81.60)	44 (18.40)	11.274	0.001
Had genital discharge in last 12 months	No	2706 (88.80)	341 (11.20)		
	Yes	588 (86.20)	94 (13.80)	3.632	0.057
Number of sex partners, including spouse, in last 12 months	1	3068 (88.70)	392 (11.30)		
	> 1	226 (84.00)	43 (16.00)	5.25	0.022
Relationship with most recent sex partner	Spouse	1159 (88.10)	157 (11.90)		
	Boyfriend not living with respondent	1045 (90.00)	116 (10.00)	86.408	0
	Live‐in‐partner	931 (90.90)	93 (9.10)		
	Others	159 (69.70)	69 (30.30)		
Wife justified asking husband to use condom if he has STI	No	956 (89.10)	117 (10.90)		
	Yes	2338 (88.00)	318 (12.00)	0.847	0.357
Drugs to avoid HIV transmission to baby during pregnancy	No	488 (94.80)	27 (5.20)		
	Yes	2806 (87.30)	408 (12.70)	23.919	0
Would buy vegetables from vendor with HIV	No	599 (95.20)	30 (4.80)		
	Yes	2695 (86.90)	405 (13.10)	34.916	0
Months ago most recent HIV test	0−12	1830 (93.00)	137 (7.00)	89.255	0
	> 12	1464 (83.10)	298 (16.90)		
Place where last HIV test was taken	Government Hospital	501 (87.40)	72 (12.60)		
	Health center	2545 (87.80)	352 (12.20)	14.944	0.001
	Others	248 (95.80)	11 (4.20)		
Total lifetime number of sex partners	1	971 (93.70)	65 (6.30)		
	2−3	1462 (89.30)	175 (10.70)	78.126	0
	> 3	861 (81.50)	195 (18.50)		
Heard of ARVs to treat HIV	No	234 (96.70)	8 (3.30)		
	Yes	3060 (87.80)	427 (12.20)	17.551	0
Knowledge and use of HIV test kits	Never heard of HIV test kits	2625 (88.40)	346 (11.60)		
	Has tested with HIV test kits	157 (87.20)	23 (12.80)	0.251	0.882
	Knows test kits but never tested with them	512 (88.60)	66 (11.40)		
Children with HIV should be allowed to attend school with children without HIV	No	488 (94.00)	31 (6.00)		
	Yes	2806 (87.40)	404 (12.60)	18.958	0
Month of most recent HIV test	1−6	1388 (88.50)	181 (11.50)		
	7−12	1363 (90.80)	138 (9.20)	31.465	0
	Don't know	543 (82.40)	116 (17.60)		
Year of most recent HIV test	2003−2020	785 (78.10)	220 (21.90)		
	2021	748 (91.20)	72 (8.80)	140.492	0
	> 2021	1761 (92.50)	143 (7.50)		
Number of HIV tests	1−2	815 (87.70)	114 (12.30)		
	3−4	1063 (89.30)	128 (10.70)	1.475	0.478
	> 4	1416 (88.00)	193 (12.00)		
Method used last sexual intercourse	No	235 (85.50)	40 (14.50)		
	Yes	3059 (88.60)	395 (11.40)	2.39	0.122

Binary logistic regression is employed as a multivariate analysis to identify the factors that substantially impact HIV and AIDS, as illustrated in Table 3. The probability of testing positive for HIV is 1.942 times higher in the South than in the North (Odds = 1.942, *p* < 0.05). The likelihood of HIV testing positive is significantly influenced by region, with the South exhibiting a higher incidence of HIV testing positive. Age was a strong predictor, with HIV risk rising steadily across age groups. Individuals aged 26–30 (OR = 3.595), 31–35 (OR = 4.744), and over 35 (OR = 7.574) had significantly higher odds of infection than those aged 15–20 (*p* < 0.05). The probability of obtaining a positive HIV test result in rural areas is 0.75 times lower than that in urban regions (odds = 0.75, *p* < 0.05). Education serves as a significant protective factor, as individuals with higher levels of schooling exhibit a markedly reduced likelihood of testing positive for HIV. The likelihood of obtaining a positive test result is 0.507 times lower for individuals with secondary education than those lacking such qualifications (odds = 0.507, *p* < 0.05). Among religious groups, only Zion affiliation was significantly associated with increased odds of HIV (OR = 1.603, *p* < 0.05). Indeed, individuals who have experienced a genital sore/ulcer within the past year have a 1.812‐fold increased likelihood of testing positive for HIV (Odds = 1.812, *p* < 0.05). Regarding relationship type, respondents with “live‐in partners” had lower odds (OR = 0.708, *p* < 0.05), whereas those in the “Others” category had substantially higher risk (OR = 2.135, *p* < 0.05). The probability of testing positive for HIV is 1.994 times higher for individuals who have had more than three sexual partners in the past year (odds = 1.994, *p* < 0.05). The increased likelihood of testing positive for individuals with multiple sexual partners is a significant risk factor for HIV transmission. Individuals tested outside government hospitals or health centers had significantly lower odds of HIV positivity (OR = 0.378, *p* < 0.05), suggesting reduced detection rates in alternative testing settings, possibly due to differences in protocols or follow‐up.

**Table 3:** Binary logistic regression determining the influential factors for the Result of HIV test in Mozambique.

Variables	B	SE	Exp(B)	95 CI for Exp(B)		*p* value
				Lower	Upper	
Region						
North						0
Central	−0.062	0.275	0.94	0.549	1.611	0.822
South	0.664	0.258	1.942	1.171	3.221	0.01
Respondent's current age						
15–20						0
21–25	0.298	0.339	1.347	0.693	2.62	0.38
26–30	1.279	0.314	3.595	1.941	6.656	0
31–35	1.557	0.316	4.744	2.552	8.818	0
> 35	2.025	0.305	7.574	4.162	13.783	0
Type of place of residence						
Urban						
Rural	−0.287	0.14	0.75	0.57	0.988	0.04
Highest educational level						
No education						0
Primary	−0.158	0.182	0.853	0.598	1.218	0.383
Secondary	−0.679	0.209	0.507	0.336	0.764	0.001
Higher	−2.049	0.443	0.129	0.054	0.307	0
Religion						
Catholic						0.296
Islamic	0.276	0.287	1.318	0.751	2.311	0.336
Zion	0.472	0.221	1.603	1.039	2.472	0.033
Evangelical/Pentecostal	0.377	0.196	1.458	0.993	2.14	0.054
Others	0.392	0.27	1.48	0.872	2.511	0.146
Socio‐economic status						
Poor						0.006
Middle	0.19	0.191	1.209	0.831	1.76	0.321
Rich	−0.315	0.177	0.73	0.516	1.032	0.075
Births in last 5 years						
No births						0.02
1	0.325	0.134	1.384	1.065	1.799	0.015
> 1	0.504	0.22	1.655	1.075	2.549	0.022
Had genital sore/ulcer in last 12 months						
No						
Yes	0.594	0.203	1.812	1.218	2.695	0.003
Number of sex partners, including spouse, in last 12 months						
1						
> 1	0.27	0.215	1.311	0.86	1.997	0.208
Relationship with most recent sex partner						
Spouse						0
Boyfriend not living with respondent	0.271	0.162	1.312	0.955	1.801	0.094
Live‐in‐partner	−0.346	0.149	0.708	0.528	0.948	0.02
Others	0.759	0.204	2.135	1.431	3.186	0
Drugs to avoid HIV transmission to baby during pregnancy						
No						
Yes	0.385	0.225	1.469	0.946	2.283	0.087
Would buy vegetables from vendor with HIV						
No						
Yes	0.882	0.274	2.417	1.414	4.131	0.001
Months ago most recent HIV test						
0−12						
> 12	0.506	0.269	1.659	0.979	2.81	0.06
Place where last HIV test was taken						
Government Hospital						0.019
Health center	−0.06	0.154	0.942	0.697	1.274	0.699
Others	−0.972	0.355	0.378	0.189	0.759	0.006
Total lifetime number of sex partners						
1						0.001
2−3	0.332	0.173	1.393	0.993	1.955	0.055
> 3	0.69	0.188	1.994	1.379	2.882	0
Heard of ARVs to treat HIV						
No						
Yes	0.607	0.402	1.835	0.834	4.037	0.132
Children with HIV should be allowed to attend school with children without HIV						
No						
Yes	0.303	0.278	1.354	0.785	2.336	0.276
Month of most recent HIV test						
1−6						0.527
7−12	−0.014	0.136	0.986	0.756	1.287	0.918
Don't know	0.168	0.159	1.183	0.866	1.616	0.29
Year of most recent HIV test						
2003−2020						0
2021	−0.82	0.164	0.44	0.32	0.607	0
> 2021	−0.639	0.259	0.528	0.317	0.878	0.014
Constant	−5.902	0.665	0.003			0
Hosmer and Lemeshow test	Chi‐square = 13.09		*p*‐value = 0.109			

The Hosmer and Lemeshow test (*p* = 0.109) indicate that the model is well‐fitted, indicating that the factors included in the model provide a reliable explanation of HIV test results.

## 4 Discussion

The primary goal of this study was to identify the factors influencing HIV and AIDS prevalence among participants in Mozambique. The findings of the study underscore regional disparities, indicating that the Southern region exhibits a higher probability of testing positive for HIV compared to the Northern region.

Our research indicates that 11.7% of the population in Mozambique is HIV‐positive, representing a significant public health concern. In contrast, the prevalence of HIV demonstrates significant variability across various countries and regions. The prevalence in Eastern China is relatively low, recorded at 0.007% [35]. It is significantly higher in Cameroon, where it stands at 5.5%, and even more pronounced in Yaoundé, which exhibits a prevalence rate of 44.4% [36]. Kenya has an HIV prevalence of 15.4%, which is higher than in other regions [37]. The United States reports an HIV‐positive prevalence rate of 10% [38], whereas the Zanzibar Archipelago, located in Sub‐Saharan Africa, exhibits a prevalence rate of 2.9% [39], exhibiting a distinct demographic and healthcare environment. The prevalence is reported to be 5.9% in Colombia [40] and 1.28% in Sierra Leone [41]. Ethiopia bears a considerable burden of HIV, with 11.9% of its population affected by the infection [42].

The results indicate that the age of the respondents has a substantial impact on the prevalence of HIV and AIDS, with an increased likelihood noted among individuals within the age ranges of 31–35 and 36–49. In Kenya, men aged 30–34 demonstrated the highest prevalence of HIV, which was recorded at 41.1%, thereby highlighting the vulnerability of this demographic group [37]. Likewise, individuals aged 26–35 in the Zanzibar Archipelago exhibited an elevated risk of contracting HIV/AIDS [38]. In Colombia, individuals aged 25–34 and those aged 35 and older exhibited more significant rates of infection compared with their younger counterparts [40]. A study conducted in Sierra Leone found that women aged 25–44 are at an enhanced risk of developing HIV, which is consistent with the pattern we have identified [41]. These findings are consistent with broader trends observed in sub‐Saharan Africa, indicating that older age groups—especially those between the ages of 30 and 34 have a greater likelihood of getting HIV and getting tested for the virus [27].

This study indicated that residing in an urban area is correlated with an elevated risk of testing positive for HIV, suggesting disparities in both exposure and access to medical care. This finding aligns with previous research conducted in Colombia, which indicated that urban residents exhibited a higher likelihood of being HIV‐positive compared to their rural counterparts [40]. Similarly, studies conducted in Sierra Leone found that those living in cities had an increased probability of being HIV positive compared to their rural counterparts [41]. Further confirming the observed trend was the increased prevalence of HIV infection among Kenyan women living in urban regions [43].

Our research indicated that higher levels of education serve as a protective factor against HIV infection, likely due to enhanced access to healthcare services and increased awareness of preventive strategies. This aligns with findings from other studies conducted in various contexts. Research conducted in Nigeria and Vietnam indicated that low levels of educational attainment constitute a significant risk factor for HIV, particularly among adults aged 15–49 and young people who engage in injecting drug use [44, 45]. Similarly, individuals residing in the Zanzibar Archipelago [39], as well as those who were illiterate or Americans who had not completed high school, showed a higher likelihood of contracting HIV [38]. HIV positivity was more common among those with only a primary education in Colombia than among those with a high school education [40], and HIV prevalence was highly associated with lower educational status in Ethiopia [42].

This study highlights the significant association between genital sores or ulcers and a higher risk of HIV infection because these conditions make it easier for the virus to spread. According to a Colombian study, women who had experienced genital sores within the previous 12 months were three times more likely to contract HIV, which is consistent with this finding [40]. Similarly, studies conducted in Ethiopia showed that a history of genital ulcers significantly increased the risk of contracting HIV [42].

This study elucidates the contribution of hazardous sexual behavior to the transmission of HIV by demonstrating that engaging in multiple sexual partnerships substantially elevates the likelihood of HIV positivity. This finding aligns with research conducted in Africa, which indicates that engaging in multiple sexual relationships is a significant behavioral factor correlated with a high prevalence of HIV among young individuals [46]. According to research conducted in Kenya, having multiple sexual partners is significantly associated to an increased risk of HIV infection [37]; women who report having four or more lifetime partners are especially at risk [43].

Our research indicates that HIV testing performed outside of governmental facilities exhibits lower detection rates, a phenomenon that may be attributable to variations in testing methodologies. This finding stands in stark contrast to prior research, which indicates that individuals prefer HIV testing in familiar medical environments such as hospitals, public health clinics, and physicians’ offices, primarily due to their accessibility and convenience [47].

These factors illustrate a lack of comprehension regarding HIV transmission and present considerable obstacles to effective prevention initiatives. This highlights the necessity for specialized educational initiatives to mitigate stigma and advance public health. Subsequent years of testing demonstrated a decline in HIV positivity rates, which can likely be ascribed to improved prevention strategies, extensive awareness campaigns, and advancements in early detection technologies. These initiatives have played a significant role in decreasing the overall prevalence by enabling the early detection of cases and reducing the occurrence of new infections.

## 5 Limitations and Strength

This study has several limitations. It relies on self‐reported data on sexual behavior, STI history, and HIV testing, which are prone to recall and social desirability bias. The cross‐sectional design also restricts the ability to infer causal relationships between identified risk factors and HIV infection. Additionally, some high‐risk or marginalized populations may be underrepresented, which could lead to an underestimation of HIV prevalence.

Despite these limitations, the study has notable strengths. It is based on recent, nationally representative data from the 2022–2023 MMDHS, providing broad coverage of both urban and rural populations. The large sample size and standardized DHS methodology enhance the reliability and comparability of the results. Moreover, the analysis incorporates a wide range of socioeconomic, demographic, cultural, biological, and behavioral variables, and applies multivariate logistic regression with appropriate model‐fit assessment, offering a comprehensive and robust evaluation of factors associated with HIV testing outcomes in Mozambique.

## 6 Conclusion

HIV infection reflects a complex interaction of socioeconomic, demographic, cultural, biological, and behavioral factors. This study examines the key determinants of HIV infection in Mozambique using a multivariate framework. The findings identify several significant predictors, including region of residence, age, place of residence, educational attainment, religious affiliation, sexual behavior, and access to HIV testing services. Individuals living in the South region, those aged 26 years and above, rural residents, and followers of the Zion religion show higher odds of HIV infection. Biological and behavioral factors—such as a history of genital sores or ulcers, engagement in live‐in or other nonspousal relationships, testing at nongovernment facilities, and having more than three lifetime sexual partners—substantially increase HIV risk. In contrast, secondary and higher education emerge as strong protective factors against infection. The results highlight persistent regional and social inequalities and the importance of education, sexual health, and service accessibility in HIV prevention. These findings can inform targeted, region‐specific, and education‐based interventions, alongside stigma reduction efforts. Future longitudinal research is needed to clarify causal pathways and to assess how changes in social norms and healthcare delivery influence HIV testing behavior and infection trends over time.

## Author Contributions

Md. Rasel Hossain, Kabir Hossain, Riyadh Hossain, and Indi Islam participated in this study through the design phase and in data acquisition, cleaning, and analysis. Md. Rasel Hossain, Indi Islam, Md. Owaliur Rahman Akanda, Nazia Akter, and Kabir Hossain participated in the analysis and interpretation of data, as well as in the drafting and revision of the manuscript. Md. Rasel Hossain participated in the oversight of the final manuscript. All authors have reviewed and approved the final manuscript.

## Funding

The authors received no specific funding for this work.

## Ethics Statement

According to the ethical principles described in the Declaration of Helsinki, the authors undertook all requisite measures to comply with the established guidelines for medical research. This study used publicly available secondary data from the 2022–2023 Mozambique Mini Demographic and Health Survey (MMDHS), conducted through The DHS Program. The MMDHS 2022–2023 followed standardized and internationally accepted ethical procedures for data collection, and informed consent was obtained from all respondents before their participation in the survey. The survey protocol received ethical approval from the relevant national ethics authorities in Mozambique as well as the Institutional Review Board (IRB) of ICF. Since the present study relied solely on anonymized secondary data and did not involve direct interaction with participants or the collection of new information, additional ethical approval was not required. However, permission to access and use the 2022–2023 MMDHS dataset for this analysis was obtained from The DHS Program.

## Consent

All respondents gave their consent before collecting the data, which was confirmed by the enumerators. All respondents provided consent for the publication of the survey results. All authors have read the manuscript and provided their consent to publish this article.

## Conflicts of Interest

The authors declare no conflicts of interest.

## Transparency Statement

The lead author Md. Rasel Hossain affirms that this manuscript is an honest, accurate, and transparent account of the study being reported; that no important aspects of the study have been omitted; and that any discrepancies from the study as planned (and, if relevant, registered) have been explained.

## Data Availability

The datasets used and analyzed during the current study are available from the corresponding author upon reasonable request. Additionally, the data was sourced from the Demographic and Health Survey (DHS) Program and is available to the public on their website [33]. The code for this research will be provided on request.
